# Molecular Epidemiology of Crimean-Congo Hemorrhagic Fever Virus in Kosovo

**DOI:** 10.1371/journal.pntd.0002647

**Published:** 2014-01-09

**Authors:** Luka Fajs, Xhevat Jakupi, Salih Ahmeti, Isme Humolli, Isuf Dedushaj, Tatjana Avšič-Županc

**Affiliations:** 1 Institute of Microbiology and Immunology, Faculty of Medicine, University of Ljubljana, Ljubljana, Slovenia; 2 National Institute of Public Health of Kosovo, Pristina, Republic of Kosovo; 3 Clinic of Infectious Diseases, Pristina, Republic of Kosovo; University of Texas Medical Branch, United States of America

## Abstract

Crimean-Congo hemorrhagic fever virus (CCHFV) is a zoonotic agent that causes severe, life-threatening disease, with a case fatality rate of 10–50%. It is the most widespread tick-borne virus in the world, with cases reported in Africa, Asia and Eastern Europe. CCHFV is a genetically diverse virus. Its genetic diversity is often correlated to its geographical origin. Genetic variability of CCHFV was determined within few endemic areas, however limited data is available for Kosovo. Furthermore, there is little information about the spatiotemporal genetic changes of CCHFV in endemic areas. Kosovo is an important endemic area for CCHFV. Cases were reported each year and the case-fatality rate is significantly higher compared to nearby regions. In this study, we wanted to examine the genetic variability of CCHFV obtained directly from CCHF-confirmed patients, hospitalized in Kosovo from 1991 to 2013. We sequenced partial S segment CCHFV nucleotide sequences from 89 patients. Our results show that several viral variants are present in Kosovo and that the genetic diversity is high in relation to the studied area. We also show that variants are mostly uniformly distributed throughout Kosovo and that limited evolutionary changes have occurred in 22 years. Our results also suggest the presence of a new distinct lineage within the European CCHF phylogenetic clade. Our study provide the largest number of CCHFV nucleotide sequences from patients in 22 year span in one endemic area.

## Author Summary

Crimean-Congo hemorrhagic fever (CCHF) is an acute, tick-borne disease with a case fatality rate of 10–30%. It is geographically the most widespread tick-borne disease in the world. In recent years there has been an increase of the disease incidence in several countries, mainly in the countries of the Balkan. The disease is also endemic in Kosovo. Since CCHF virus is very genetically diverse we aimed to determine the genetic variability of the virus in Kosovo in the span of 22 years. We obtained the largest number of patient derived nucleotide sequences and found great genetic variability which has been more or less stable during the 22 year period. Our results also suggest that significant changes in viral population occur in different years. We show that ecological factors such as temperature could play a role in the composition of the viral population.

## Introduction

Crimean-Congo hemorrhagic fever (CCHF) is an acute tick-borne zoonotic disease which is characterized by a fulminant and often hemorrhagic course of disease with the case fatality rate of 10–50%. Causative agent is the Crimean-Congo hemorrhagic fever virus (CCHFV) which belongs to the *Nairovirus* genus in the family *Bunyaviridae*. CCHF is the most widespread tick-borne disease in the world with cases reported in a number of countries in Africa, Asia, Middle East and southeastern Europe. Geographical distribution is closely linked to the presence of the primary vectors, ticks of the genus *Hyalomma* [1]. CCHFV genome consists of three single-stranded negative-sense RNA segments: small (S), medium (M) and large (L) [1], [2]. Genetic analyses of all three genomic segments have shown that CCHFV exhibits a high level of genetic variability ranging from 20% (S segment), 22% (L segment) to 31% (M segment). Genetic variability correlates with the geographical spread of the virus. Namely, phylogenetic analyses of the S segment have shown that geographically separated viral isolates cluster in roughly six clades: two European, three African and one Asian [3]. Genetic variability of CCHFV was also demonstrated within several geographical regions. For example, Ozkaya et al. (2010) have shown existence of local topotypes of CCHFV in Turkey [4] while Aradaib et al. (2011) have found the presence of several variants of CCHFV in Sudan [5].

CCHF is endemic in Kosovo. The first reports of CCHF in Kosovo date back to 1957, when a family outbreak resulting of eight fatal cases, was described [6]. Based on the records of the Institute of Public Health of Kosovo, from 1995 to August 2013, 228 cases of CCHF have been reported in Kosovo, with the mortality rate of 25.5%. There is limited information about CCHFV genetic diversity in Kosovo despite the long presence of CCHFV infections in this area [7], [8], [9]. The aim of our study was to investigate the genetic variability of CCHFV from patients in Kosovo in a time span of 22 years in order to determine the spatio-temporal characteristics of CCHFV in this highly endemic area.

## Methods

### Patient samples

For the purpose of the study, we included 89 serum samples of Real-Time RT-PCR confirmed CCHF patients from Kosovo, hospitalized from 1991–2013. Serum samples were periodically received from the National Institute of Public Health of Kosovo, Republic of Kosovo for confirmatory diagnostics and further analyses. Samples were processed as previously described [10]. The study was retrospective therefore we did not obtain additional informed consent from the patients. Instead, the research was approved by the National Medical Ethics Committee of the Republic of Slovenia. We followed the principles of the Helsinki Declaration, the Oviedo Convention on Human Rights and Biomedicine, and the Slovene Code of Medical Deontology. All human samples were anonymized and no additional sample was taken for the purpose of the study.

### RNA isolation and RT-PCR

Total RNA from serum samples between years 1991–2009 was extracted using Trizol LS Reagent (Invitrogen Life Technologies) according to the manufacturer's instructions. Total RNA from serum samples between years 2010–2013 was extracted using QIAamp Viral RNA Mini Kit (Qiagen) according to the manufacturer's instructions. RT-PCR amplification of the complete S segment was performed as described by Deyde et al. [3]. RT-PCR was performed using the SuperScript III One-Step RT-PCR System with Platinum Taq High Fidelity (Invitrogen Life Technologies) according to the manufacturer's instructions. Nested PCR was performed using primer pair CCHF SORF-F (5′-GCCATGGAAAACAAGATCGAGG-3′) and CCHF SORF-R (5′-AGTTCTAGATGATGTTGGCAC-3′), yielding a PCR product of 1,456 bp which represents the complete coding region of the CCHF N protein. Nested PCR was performed using KOD Xtreme Hot Start DNA Polymerase (Novagen, EMD4Biosciences) according to the manufacturer's instructions. Nested PCR cycling conditions were as follows: initial denaturation at 94°C for 2 minutes, followed by 40 cycles of denaturation at 98°C for 10 seconds, primer annealing at 60°C for 30 seconds and elongation at 68°C for 1 minute and 30 seconds. Additionally, a 536 bp fragment (primers CCHF F2/R3) or a 260 bp fragment (primers CCHF F3/R2) of the S segment was amplified as described by Rodriguez et al. [11] if the amplification of the 1,456 bp fragment was not successful. Partial M segment nucleotide sequences were obtained as described previously [12].

### Sequencing and nucleotide sequence analysis

PCR products were purified with the Wizard SV Gel and PCR Clean-Up System (Promega), sequenced using the BigDye Terminator 3.1 Cycle sequencing kit (Applied Biosystems) and analyzed with the 3500 Genetic Analyzer (Applied Biosystems).

Nucleotide sequences were assembled and edited using CLC Main Workbench software (CLC bio, Denmark). At least two-fold read coverage was obtained for all sequences. Sequences were aligned in MEGA version 5 [13] using Muscle algorithm. Nucleotide sequences were deposited to the GenBank database (accession numbers KC477779-837, KF039932-83, KF595127-49). Nucleotide substitution model was selected based on Akaike's information criterion (AIC) in jModelTest, version 0.1.1 [14]. The general time-reversible model with gamma-distributed rate variation (GTR+G) was employed for phylogenetic analyses of the CCHF S segment. Bayesian phylogenetic analyses were performed in MrBayes 3.2 [15] and Tracer version 1.5 [16]. Four independent Markov Chain Monte Carlo (MCMC) runs of four chains each consisting of 10,000,000 generations were run to ensure effective sample sizes (ESS) of at least 1000. Phylogenetic analysis of the M segment sequences was performed in MEGA5: Molecular Evolutionary Genetics Analysis [17]. The TN92 model with gamma-distributed rate variation was used for the analysis. Maximum clade credibility trees were depicted using FigTree version 1.3.1 [16]. Evolutionary rates and calculation of the time of the most recent common ancestor (tMRCA) were determined for the larger S segment sequences. We estimated the evolutionary rates using a MCMC method implemented in BEAST 1.8.0 [16] with a relaxed molecular clock (under the GTR+G+I model of nucleotide substitution) and a piecewise-constant Bayesian skyline plot as a coalescent prior. Priors were selected according to Zehender et al. [18]. The chains were conducted until reaching ESS>200 and sampled every 10,000 steps. Trees were summarized in a maximum clade credibility tree after a 10% burnin using Tree Annotator 1.8.0 [16]. Mean evolutionary rates and tMRCA were calculated in TreeStat 1.8.0 [16].

## Results

**Figure 1:**
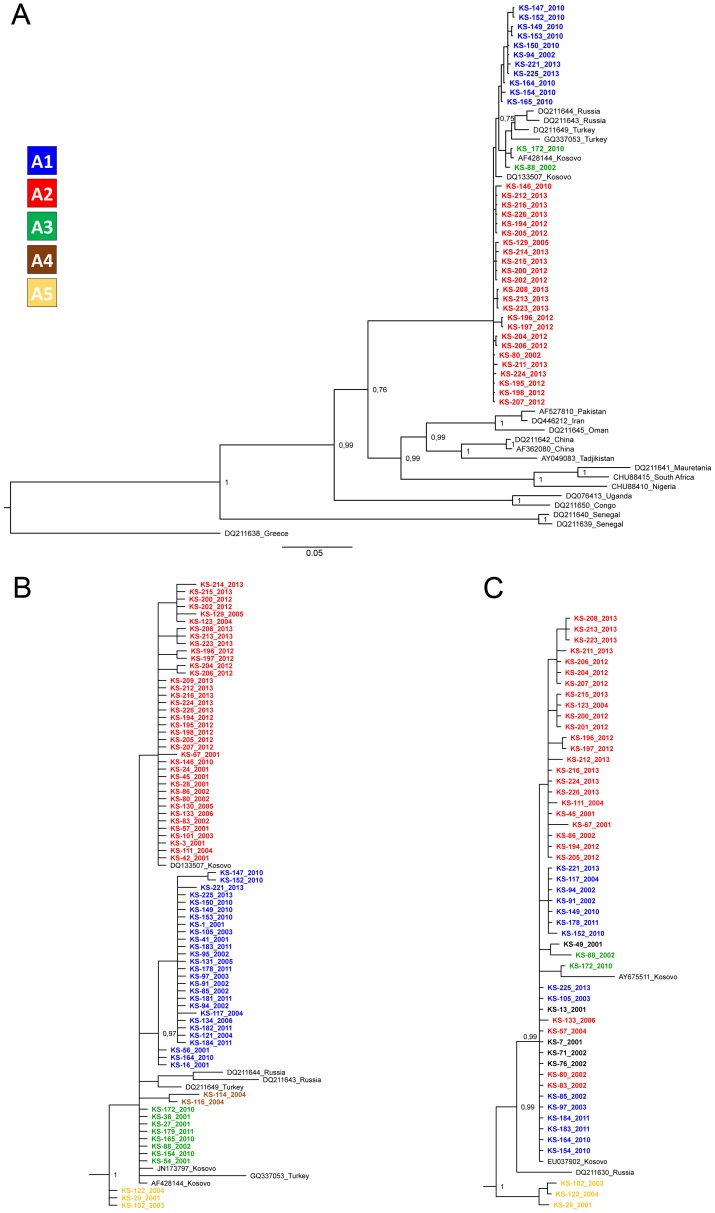
Bayesian phylogenetic analysis of the A. 1019 bp fragment of the CCHFV S segment, B. 389 bp fragment of the CCHFV S segment, C. partial M segment sequences. Sequences from patients in Kosovo are designated with KS, followed by patients' ID and year of hospitalization. GenBank accession numbers of reference sequences are shown alongside CCHFV strain origin. Branch labels represent posterior probabilities. Designations A1–A5 represent the assigned phylogenetic clusters based on the analysis of the 389 bp fragment. Samples in black type in the M segment analysis did not have a representing S segment sequence.

We obtained 37 partial CCHFV S segment sequences (1019 bp) from patients hospitalized in 2002 (n = 3), 2005 (n = 1), 2010 (n = 10), 2012 (n = 11) and 2013 (n = 12). All sequences clustered in the European CCHF genetic lineage V, along with previously published CCHFV sequences from Kosovo (Figure 1A). Overall identity of the sequences ranged from 98.8–100% and we detected three amino acid changes; S272N (present in samples KS153 and KS149), K316R (present in samples KS208, KS213 and KS223) and V327I (present in samples KS172 and KS88)(amino acid positions are numbered relative to the nucleoprotein sequence of CCHFV strain Kosovo Hoti, accession number: AAZ32529). CCHFV sequences clustered in roughly three groups designated A1–A3 (Figure 1A). We estimated a mean evolutionary rate of 2.76×10^−4^ substitutions/site/year and the mean tMRCA for the root of 729.4 years ago.

We then analyzed a shorter fragment of the S segment (389 bp), because we had more sequences available. We obtained 79 nucleotide sequences from patients hospitalized in 2001 (n = 15), 2002 (n = 8), 2003 (n = 4), 2004 (n = 7), 2005 (n = 3), 2006 (n = 2), 2010 (n = 10), 2011 (n = 6), 2012 (n = 11) and 2013 (n = 13). Overall identity of the sequences ranged from 98.5–100%. All sequences clustered in the European genetic lineage V and were distributed in 5 genetic groups (A1–A5). The latter phylogenetic analysis was comparable to the previous one, although some resolution was lost. Samples KS-154 and KS-165, which clustered in group A1 in the previous analysis were miss-assigned to group A3.

The most divergent sequences clustered into group A5. This cluster was also most divergent compared to other sequences in the European genetic lineage V (maximum nucleotide distance within the European genetic lineage V was 2.9, that is to the Turkish GQ337053 sequence).

We additionally obtained 4 partial S segment sequences (220 bp) from patients hospitalized in 1991 (n = 3) and 1992 (n = 1). These sequences were not included in the previous phylogenetic analysis because they were too short. However, clustering into groups A1–A5 can be distinguished by analysis of mutational profiles of four nucleotide changes: 343T/C, 496C/A, 304C/T or 520A/G and 220T/C or 550T/C (nucleotide positions are numbered relative to the complete S segment sequence of CCHFV strain Kosovo Hoti, accession number: DQ133507). Thereby we were able to assign two sequences from 1991 to group A2, while the two other sequences could not be definitely assigned (sequences could be assigned to either group A3 or A4).

In order to further support our findings, we sequenced 431 bp of CCHFV M segment. We obtained 50 partial M segment sequences. Overall identity of the sequences ranged from 95.2–100%. In general we observed three distinct phylogenetic groups; A1, A2 and A5 (Figure 1C). Several sequences could not be assigned to any of the observed groups due to the low resolution of the phylogenetic analysis. Despite several attempts we could not obtain longer M segment sequences from these samples due to low sample volumes and low viral loads. Therefore, we could not obtain a phylogenetic tree with higher resolution.

**Figure 2:**
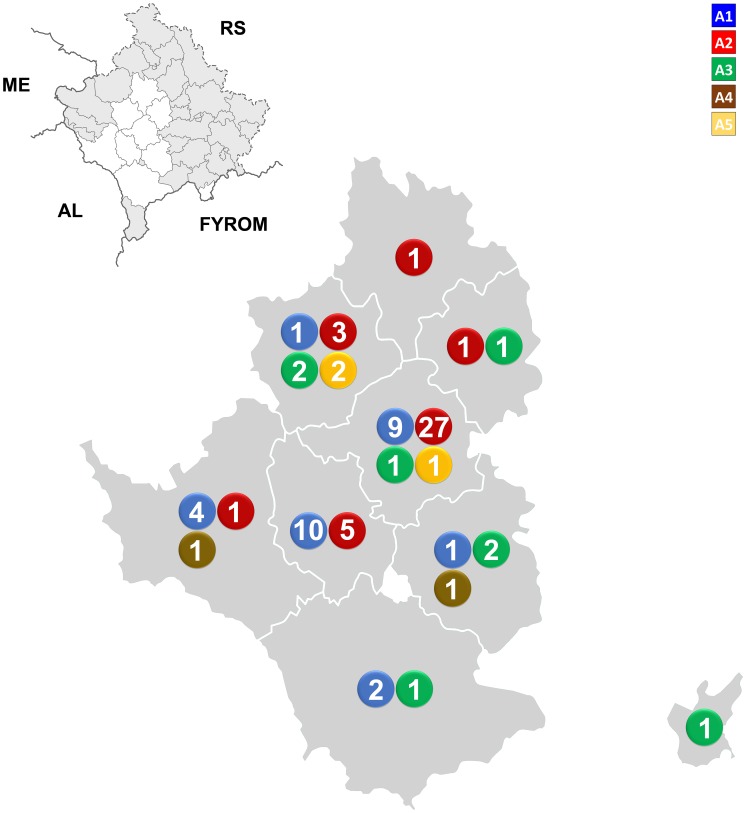
Correlation of geographical and phylogenetic clustering of CCHFV sequences in Kosovo. Sequence abundances were plotted on the map of Kosovo. The numbers represent the number of obtained sequences. Designations A1–A5 represent the assigned phylogenetic clusters. RS = Republic of Serbia, ME = Montenegro, AL = Albania, FYROM = Former Yugoslav Republic of Macedonia.

Next, we wanted to determine the geographical distribution of the sequences. Each phylogenetic cluster was plotted on the map of Kosovo with respect to the grouping from the 389 bp S segment phylogenetic analysis. As is seen in Figure 2 sequences are evenly distributed throughout the studied area. The two most abundant phylogenetic groups (A1 and A2) are present in almost all studied municipalities. However, sequences from group A1 are present in southern parts in greater abundance than in the northern parts and vice versa for group A2. The number of sequences we obtained is comparable to the incidence of CCHF in each municipality. On average we sequenced approximately 50% of total confirmed cases in each municipality. Therefore our results portray a realistic picture of the distribution of viral variants in the endemic area. Sequences from the most divergent phylogenetic group (A5) grouped in two neighboring municipalities in central Kosovo. No obvious ecological or geographical barriers are present in this area which could explain the constrained geographical distribution of the variants.

We did not observe any temporal correlation to the phylogenetic clustering. From 2001 to 2010 the two major phylogenetic groups (A1 and A2) occurred in similar abundances. However, significant shifts in abundances of the two groups occurred in the following years. In 2011, 80% confirmed patients were infected with A1 virus variant (and 20% with A3). On the contrary, in 2012 we detected the A2 virus variant alone (we sequenced 92% confirmed CCHF cases). In 2013, again both A1 and A2 variants were present (9% and 50% confirmed cases, respectively).

## Discussion

CCHFV is a genetically diverse virus. It groups into several genetic clades which correlate to the geographic origin to some extent. This correlation is most profoundly seen in the phylogenetic analyses of the viral S segment. The virus groups into seven phylogenetic clades: 2 European, 3 African and 2 Asian [19]. Great genetic diversity of CCHFV has also been shown within each phylogenetic clade in different extents [20]. Several viral variants were detected also within particular endemic areas [4], [5], [21], [22], [23], [24]. Furthermore, Ozkaya et al. [4] showed that same viral variants also cluster together geographically.

CCHFV is an important causative agent of disease in Kosovo. Due to the high number of CCHF cases in relation to the small size of the endemic area and the long history of CCHF in Kosovo, this area represents an interesting model for studies of viral evolution and genetic variability. The aim of our study was to expand the limited knowledge about the genetic variability of CCHFV in Kosovo. We wanted to obtain partial genome sequences directly from patient serum samples without prior cultivation or cloning in a time span of 22 years. We wanted to determine if there is any geographical clustering of the viral variants and if there were any significant temporal genetic changes.

The results of our study revealed that several viral variants are present within the endemic region in Kosovo. Overall nucleotide sequence divergence (2%) is in the scope with previous reports [20]. At least three major phylogenetic groups were formed based on the analysis of a larger portion of the viral S segment. These groups could also be discriminated in the analysis of a smaller S segment fragment. This analysis revealed the presence of 5 distinct phylogenetic clades. Previous report from Turkey described the detection of two genetic variant, or topotypes. Given the fact that the studied area in this report was at least 10 times larger than ours, implies that the overall genetic diversity of CCHFV in Kosovo is very high [4]. This difference can be attributed to several factors. The first is the number of sequenced patients, or rather the proportion of sequenced patients. In our study we sequenced 59% confirmed patients (a total of 168 confirmed cases from 2001 to 2013), a proportion that is significantly higher than in previous reports. Length of CCHF presence in an endemic area is also important. The first reports of CCHF in Kosovo date back to 1957, with several sporadic or epidemic years until present. In Turkey however, these reports are scarce and the disease has gained recognition only recently in the last ten years. Our results also suggest that the disease has been present in Kosovo for a long time and that the virus population has been more or less stable during the last 22 years. Variant analysis of nucleotide sequences obtained from patients in years 1991 and 1992 revealed that A2 group has been present throughout the whole period, whilst the existence of A1 group could not be confirmed. We estimated a mean evolutionary rate of 2.76×10^−4^ substitutions/site/year which is in concordance to the estimated evolutionary rate reported in a recent, comprehensive report of whole S segment sequences by Zehender et al. (2.96×10^−4^ substitutions/site/year) [18]. Similarly, we show that the most probable location of the MRCA in Europe was Russia and that the virus was introduced in Kosovo somewhat 50 years ago which coincides with the first reports of the disease in Kosovo in 1957 [25] (Figure S1).

With regard to the temporal changes in virus population we observed changing dynamics of viral variant abundances from 2011 to 2013. From 2001 to 2011 we steadily detected both major phylogenetic groups (A1 and A2) regardless of the number of cases in each year. However in 2011 we detected only the A1 groups (out of the two major groups) and in 2012 we detected only the A2 group. Such a rapid change in relative abundances is somewhat surprising. We could not determine any link with the geographic distribution of the cases nor to any demographic changes in this period. These observations lead us to believe that the underlying cause for the shifts probably lie in the ecology of the disease. There is limited ecological data for Kosovo available, so we could not perform an in-depth analysis. What we have found is that average yearly temperatures in 2010 and 2011 were below average and that average minimum temperatures in 2012 were below average. Data suggest that weather conditions in 2010–2013 changed in relation to previous years. Since climate greatly influences both the vector and the reservoir of the disease, the changing climate patterns could explain the changes in the viral populations. Our results suggest that relative abundances of viral variants are dynamic and are prone to great variations and that ecological factors can play a role in shaping these populations.

Of note regarding genetic diversity is also the cluster of three sequences in clade A5, which is separated from all other sequences present in Kosovo. Furthermore, our results also suggest that this lineage is also significantly different from other sequences in the European CCHFV phylogenetic clade. Spatial analysis of these sequences revealed that all three patients from whom the viral sequences were derived were infected in nearby municipalities, separated no more than 20 km apart. In combination with the temporal analysis it is also evident that the viral variant was present in the area for at least three years. This geographical limitation of the A5 phylogenetic clade is surprising since no obvious ecological and geographical obstacles are present in the area. A greater effort to obtain sequences in this region should be implemented to resolve this issue.

Spatial analysis of other phylogenetic clades observed within Kosovo patients did not reveal a clear geographical separation of the major clades. On the other hand, further inspection of the geographical clustering revealed that sequences from the phylogenetic clade A1 clustered more in the southern part of Kosovo, while sequences from clade A2 clustered more in the northern part of Kosovo.

Our study provides the first insight into the genetic variability of CCHFV in patients from Kosovo. It provides the largest set of patient derived CCHFV sequences within one geographical area in the span of 22 years. Our results reveal great genetic variability of CCHFV in Kosovo. This diversity is exemplified when we take into account the size of the studied area. Presence of several viral variant and the observed limited evolutionary changes in 22 years suggest that CCHFV has been present in Kosovo for a long time. Our results also suggest that the population of viral variants is prone to significant changes in different endemic years. Further studies are however needed to determine the factors responsible for these changes.

## Supporting Information

Figure S1 Midpoint rooted maximum clade credibility tree showing Bayesian phylogenetic analysis of the 1019 bp fragment of the CCHFV S segment estimated with a relaxed molecular clock (under the GTR+G+I model of nucleotide substitution) and a piecewise-constant Bayesian skyline plot as a coalescent prior. Designations A1–A3 represent the assigned phylogenetic clusters of sequences from Kosovo. Designations I–VI represent the CCHFV phylogenetic clades as described by Deyde et al. [19].
(TIF)
