# Supplementary figures and images for: Molecular Epidemiology of Crimean-Congo Hemorrhagic Fever Virus in Kosovo
Source: PLoS Negl Trop Dis. 2014 Jan 9;8(1):e2647. doi: 10.1371/journal.pntd.0002647 (PMC3886908; doi:10.1371/journal.pntd.0002647)

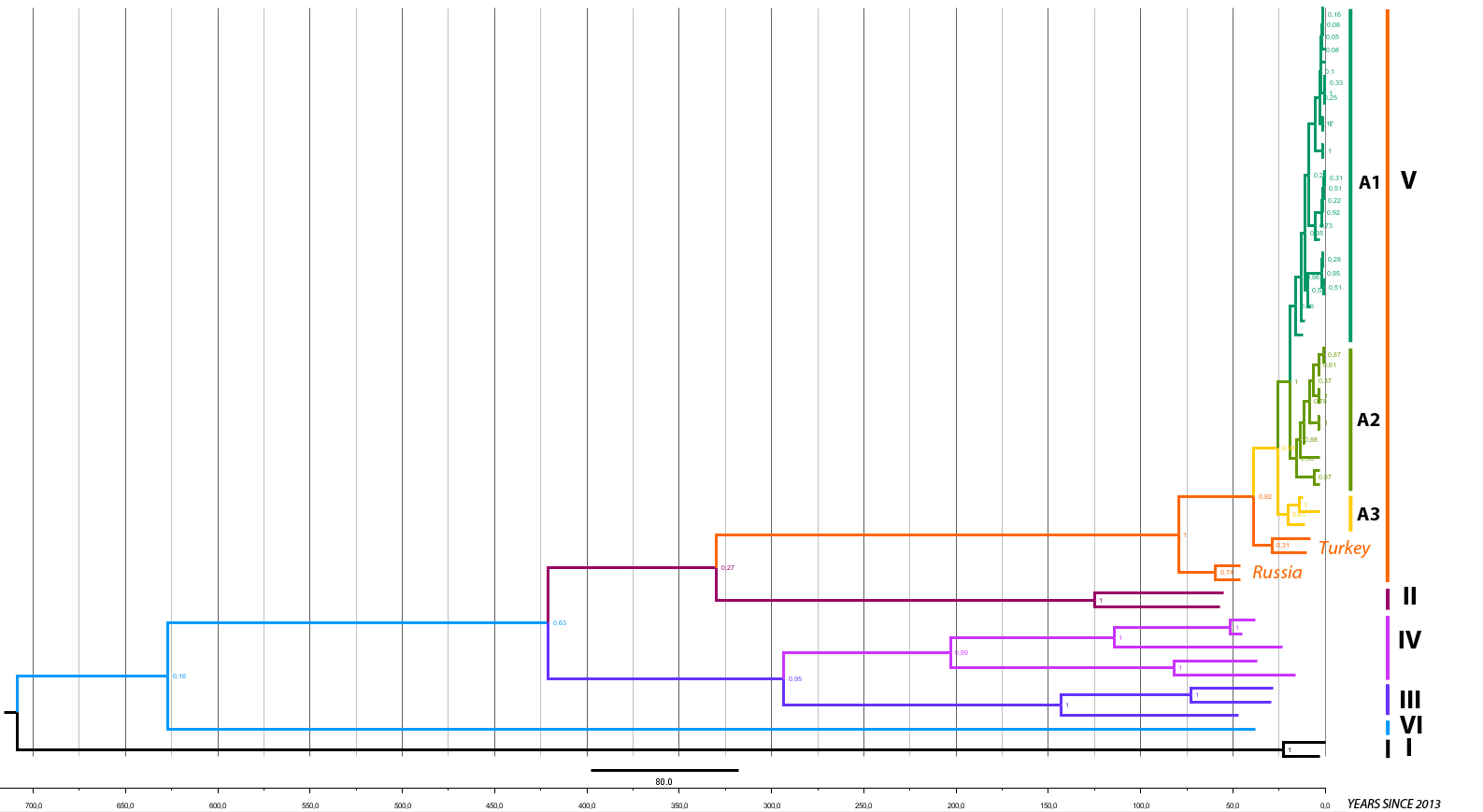

Supplement: Figure S1 — Midpoint rooted maximum clade credibility tree showing Bayesian phylogenetic analysis of the 1019 bp fragment of the CCHFV S segment estimated with a relaxed molecular clock (under the GTR+G+I model of nucleotide substitution) and a piecewise-constant Bayesian skyline plot as a coalescent prior. Designations A1–A3 represent the assigned phylogenetic clusters of sequences from Kosovo. Designations I–VI represent the CCHFV phylogenetic clades as described by Deyde et al. [19]. (TIF) [file pntd.0002647.s001.tif]
